# Peptidylarginine Deiminase 4 Deficiency Suppresses Neutrophil Extracellular Trap Formation and Ameliorates Elastase-Induced Emphysema in Mouse Lung

**DOI:** 10.3390/ijms26125573

**Published:** 2025-06-11

**Authors:** Megumi Katsumata, Jun Ikari, Akira Urano, Eiko Suzuki, Kazuto Kugou, Yoshinori Hasegawa, Koichiro Tatsumi, Takuji Suzuki

**Affiliations:** 1 Department of Respirology, Graduate School of Medicine, Chiba University, Chiba 260-8670, Japan; 2 Department of Applied Genomics, Kazusa DNA Research Institute, Kisarazu 292-0818, Japan; 3 Synergy Institute for Futuristic Mucosal Vaccine Research and Development, Chiba University, Chiba 260-8670, Japan

**Keywords:** chronic obstructive pulmonary disease (COPD), neutrophil extracellular traps (NETs), peptidylarginine deiminase 4 (PAD4), RNA sequencing

## Abstract

Neutrophil extracellular traps (NETs) are associated with the extracellular release of nuclear chromatin decorated with cytoplasmic proteins. Excessive release of NETs has been reported in chronic lung diseases, including chronic obstructive pulmonary disease (COPD). However, the role of NETs in the pathogenesis of COPD remains unclear. Peptidylarginine deaminase 4 (PAD4) contributes to NET formation. Therefore, in an elastase (ELS)-induced emphysema mouse model, we examined the role of PAD4 using *Padi4* gene knockout (KO) mice. First, we confirmed that ELS induced NET formation in the parenchyma of the lungs. PAD4 deficiency suppressed ELS-induced NET expression and tended to ameliorate the lung tissue injury. The cellular profile of bronchoalveolar lavage fluid (BALF) did not differ between the two groups. Additionally, PAD4 deficiency ameliorated emphysema and apoptosis in lung cells. Finally, we examined the effects of PAD4 on comprehensive gene expression signatures using RNA sequencing. Enrichment analysis of the transcriptomic data revealed that the expression of several genes associated with COPD pathogenesis was altered in the KO mice. Overall, the results suggest that PAD4 deficiency improves NET formation and emphysema in the lungs; this pathway can be a potential therapeutic target for the treatment of COPD.

## 1. Introduction

Chronic Obstructive Pulmonary Disease (COPD) is an airway inflammatory disease primarily caused by smoking. Multiple etiological factors, including oxidative stress, mainly associated with reactive oxygen species (ROS) [1,2,3], protease/antiprotease imbalance [4], inflammatory cells, and inflammatory mediators released from them [5], potentially contribute to COPD. Patients with COPD exhibit elevated neutrophil counts in lung tissue and airways, which are associated with the severity of this disease [6,7,8], suggesting that the neutrophilic inflammation contributes to COPD pathogenesis.

Large amounts of neutrophil elastase (NE) present in the cytoplasm of neutrophils are extracellularly released during inflammation. NE is involved in endothelial injury [9], emphysema formation [10], and apoptosis [11]. However, the precise role of neutrophils in the pathogenesis of COPD remains unclear.

Neutrophil extracellular trap (NET) formation is associated with the extracellular release of nuclear chromatin decorated with cytoplasmic proteins [12]. In nuclei of normal cells, DNA wraps around histones; moreover, neutrophils contain proteolytic enzymes, such as myeloperoxidase (MPO) and NE, in cytoplasmic granules. When neutrophils are activated by infection or other stimuli to initiate NET formation, histones are initially citrullinated via peptidylarginine deaminase 4 (PAD4) in the nucleus [13]. Consequently, the strong positive charge of histones is reduced, weakening the histone–DNA bond and causing de-condensation. Additionally, neutrophils produce reactive ROS, which facilitates the translocation of intracellular proteins, such as MPO and NE, from the granules to the nucleus and their binding to chromatin. The resulting decondensed chromatin, decorated with intranuclear proteins and histones, is released from the cell as a NET [14].

NETs are associated with an efficient immune response to infection, which protect cells against pathogen dissemination through reticular structures and a localised elevation of antimicrobial protein levels. However, the excessive release of NETs is associated with the pathogenesis of several diseases, including autoimmune and inflammatory disorders, thromboembolism, and chronic lung diseases [15,16,17]. Previously, we reported that the deficiency of PAD4 ameliorates bleomycin (BLM)-induced formation of NETs and lung fibrosis in mice [18]. NETs are also involved in the pathogenesis of COPD; they are found in the sputum of patients in stable and exacerbation phases of COPD [19,20]. Furthermore, the number of NETs negatively correlates with respiratory function [20]. However, the role of NETs in the pathogenesis of COPD remains unclear.

Generally, DNase treatment effectively supports the degradation of NET DNA scaffolds; however, the post-DNase digestion fate of NET proteins, especially histones, remains unclear. For instance, Saffarzadeh et al. demonstrated that DNase degradation of NETs is insufficient in eliminating their cytotoxic effects, suggesting that histone proteins potentially mediate most of the tissue damage associated with NETs, even after DNA digestion [21]. Hence, preventing NET formation rather than degrading them might be an effective approach for developing selective inhibitors targeting NETs. Moreover, PAD4 plays an important role in the NET formation [22]. In addition, PAD4 expression is induced by smoking [23]. Therefore, we hypothesised that the inhibition of PAD4 can be an effective way to suppress NET formation.

In this study, we aimed to determine whether NETs were induced in the lungs of mice with elastase (ELS)-induced emphysema. Moreover, we explored whether PAD4 deficiency alleviates ELS-induced NET and emphysema formation using PAD4 knockout (PAD4-KO) mice. We used RNA sequencing to elucidate the effects of PAD4 on the comprehensive gene expression signatures in ELS-induced lung inflammation and emphysema.

## 2. Results

### 2.1. PAD4 Deficiency Suppresses NET Formation and Tends to Ameliorate ELS-Induced Injury in the Lung on Day 1

**Figure 1:**
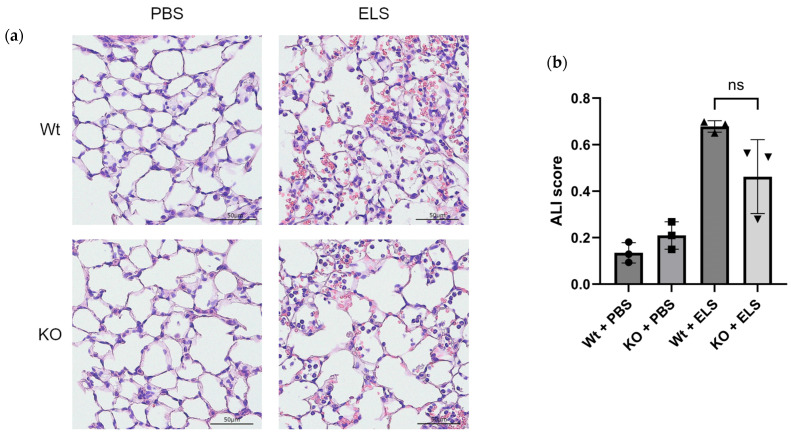

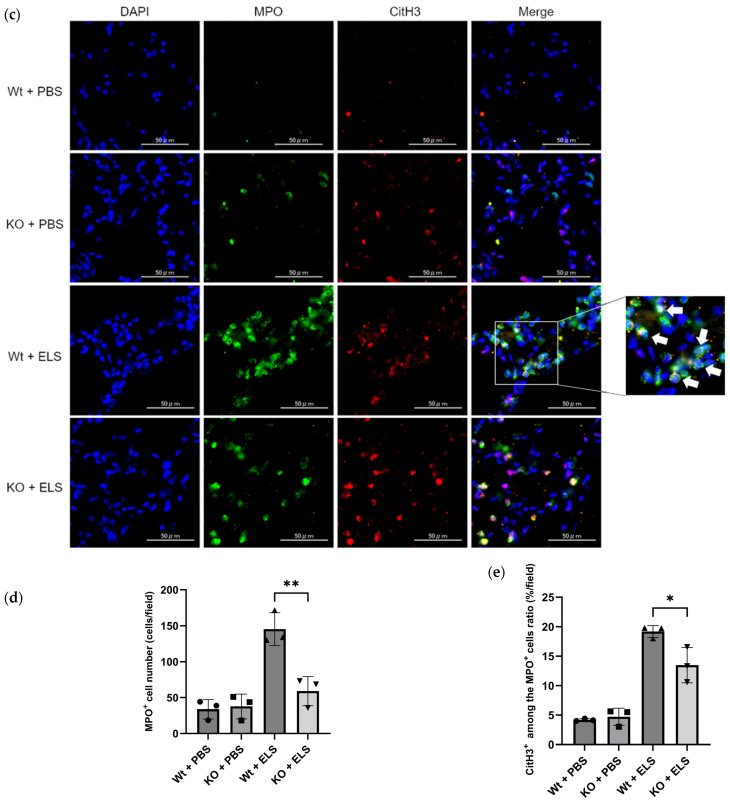
PAD4 deficiency suppresses ELS-induced neutrophil migration and expression of NETs in the parenchyma of the lung. Wt and KO mice received ELS or PBS intratracheally. Histopathology and immunofluorescence of the lung were analysed on day 1. (**a**) HE staining of each group. Scale bars, 50 µm. (**b**) ALI score (*n* = 3). (**c**) immunofluorescent staining of each group. Scale bars, 50 µm. (**d**) MPO^+^ cell number (*n* = 3). (**e**) CitH3^+^ among the MPO^+^ cells ratio (*n* = 3). ns, not significant, * *p* < 0.05, ** *p* < 0.01.

First, we examined whether PAD4 deficiency alleviated ELS-induced lung injury on day 1. Histopathological analysis revealed that the wild-type (Wt) + ELS group showed more significant haemorrhage, formation of intra-alveolar fibrin fibre and hyaline membrane, and hyaline thrombi in the lung parenchyma than the KO + ELS group (Figure 1a). The acute lung injury (ALI) scoring [24] revealed a trend towards suppressed lung injury in the KO + ELS group compared to the Wt + ELS counterpart (Figure 1b).

We explored whether ELS induced NET formation and whether PAD4 was involved in this process; we identified the presence of NETs using MPO and citrullinated histone H3 (CitH3) immunostaining following a previously reported method [25]. In Wt mice, ELS instillation significantly increased the MPO^+^ cells and NET expression (MPO^+^ and CitH3^+^) in the lung parenchyma (Figure 1c, arrow). The number of MPO^+^ cells and the frequency of CitH3^+^ cells among the MPO^+^ cells were significantly decreased in the KO group (Wt + ELS vs. KO + ELS; 145.3 ± 18.7 cells vs. 59.0 ± 16.7 cells, *p* = 0.0082; 19.2 ± 0.82% vs. 13.5 ± 2.44%, *p* = 0.035) compared to the Wt + ELS counterparts (Figure 1d,e). These results suggested that PAD4 deficiency suppressed ELS-induced NET expression and is associated with the amelioration of lung tissue injury.

### 2.2. PAD4 Deficiency Did Not Affect the Cellular Profile of the Bronchoalveolar Lavage Fluid (BALF)

**Figure 2:**
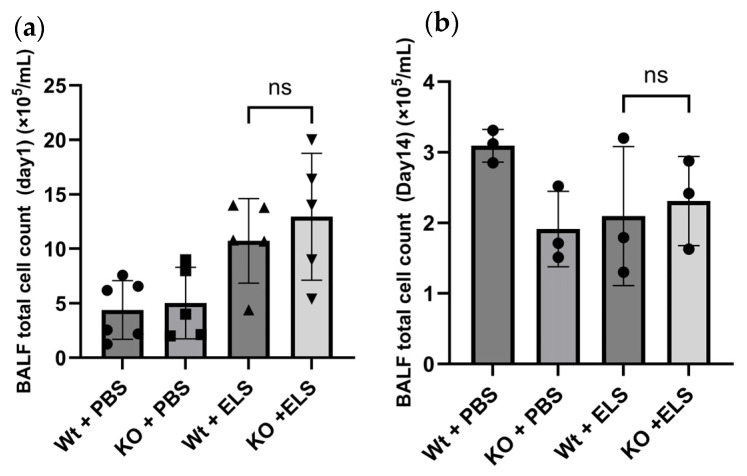
PAD4 deficiency did not affect the cellular profile of the BALF after the intratracheal administration of elastase (ELS) or phosphate-buffered saline (PBS) in the wild-type (Wt) and *Padi4* gene-knockout (KO) mice. (**a**) The total cell number in BALF on day 1 (*n* = 5–6); (**b**) the total cell number in BALF on day 14 (*n* = 3); ns, not significant.

We investigated whether PAD4 deficiency influences the inflammatory cell influx to the alveolar region caused by ELS instillation through bronchoalveolar lavage (BAL) on day 1 and day 14. On day 1, ELS treatment increased the total cell count and neutrophil fraction in both groups (Figure 2a). Moreover, macrophage fractions were suppressed in both ELS-treated groups on day 1; however, no significant difference between the Wt + ELS and KO + ELS groups was detected. Cell counts and neutrophil fractions were almost normalised in the Wt + ELS and KO + ELS groups 14 days post-ELS instillation (Figure 2b); no significant differences were observed between groups. Hence, PAD4 deficiency did not alter the inflammatory cellular influx to the alveolar region.

### 2.3. PAD4 Deficiency Suppresses the Extent of Emphysema

**Figure 3:**
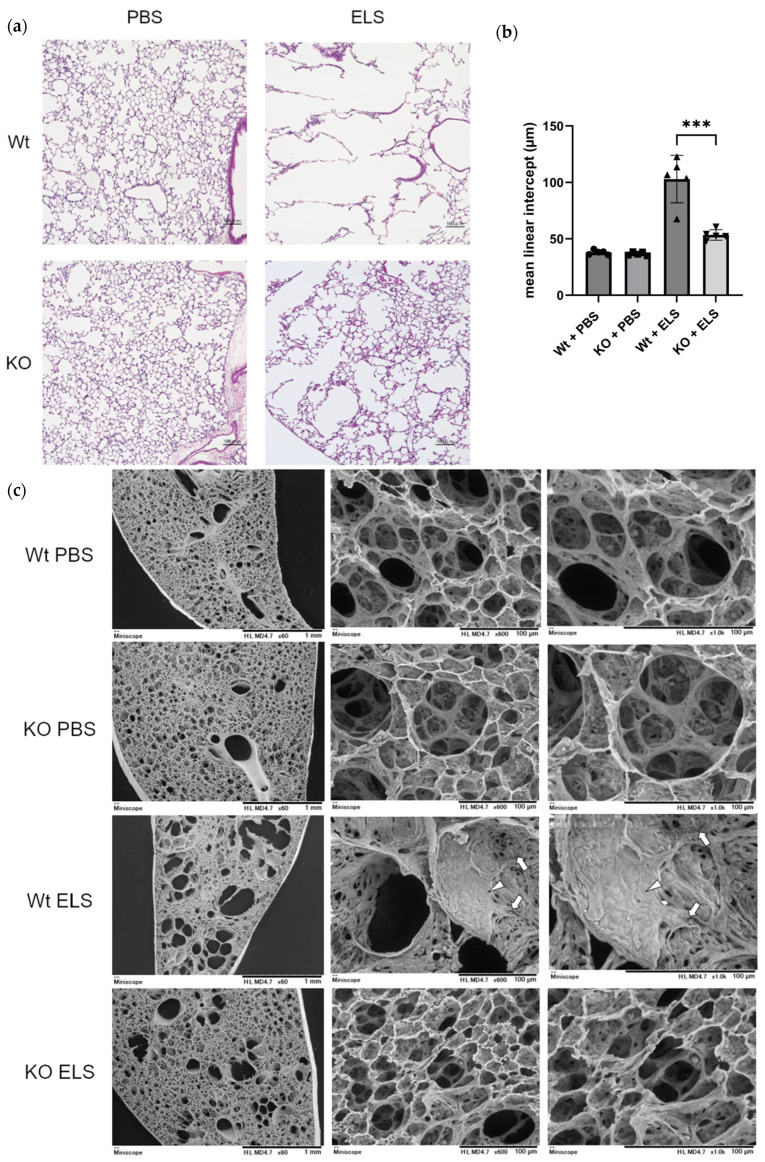
PAD4 deficiency suppresses the extent of emphysema. Wt and KO mice received ELS or PBS intratracheally. (**a**) HE staining. Scale bars, 100 µm. (**b**) Calculation of mean linear intercept (Lm) (*n* = 5). (**c**) SEM images of lung tissues on day 14. Left; ×60, scale bar, 1 mm, middle; ×600, scale bar 100 µm, right; ×1000, scale bar 100 µm. *** *p* < 0.001.

We examined whether PAD4 deficiency alleviated ELS-induced lung emphysema. HE staining of lung tissue sections revealed significantly reduced pulmonary emphysema in the lungs of PAD4-KO mice than in Wt mice after ELS instillation (Figure 3a). The mean linear intercept (Lm) was significantly decreased in KO mice compared to that in Wt mice (Wt + ELS vs. KO + ELS; 103.0 ± 18.8 µm vs. 53.5 ± 4.1 µm; *p* = 0.009) (Figure 3b).

Scanning electron microscopic (SEM) images of lung tissue revealed significant alveolar expansion in the Wt + ELS group on day 14. Furthermore, the alveolar pores disappeared from the walls of the expanded alveoli, the unevenness of the alveolar wall due to capillaries could not be confirmed, and the surface was covered with a single layer of flat squamous epithelial cells (Figure 3c, arrow). Additionally, at the edge of the same area, shrunken epithelial cells and those having extended cell processes were observed (Figure 3c, arrowhead). Although a small number of alveolar macrophages were observed in the Wt + ELS group, no infiltration of other inflammatory cells was detected; therefore, the SEM analysis reflected scarring in the Wt + ELS group due to emphysema.

### 2.4. PAD4 Deficiency Suppresses the Extent of Apoptosis in the Lung

**Figure 4:**
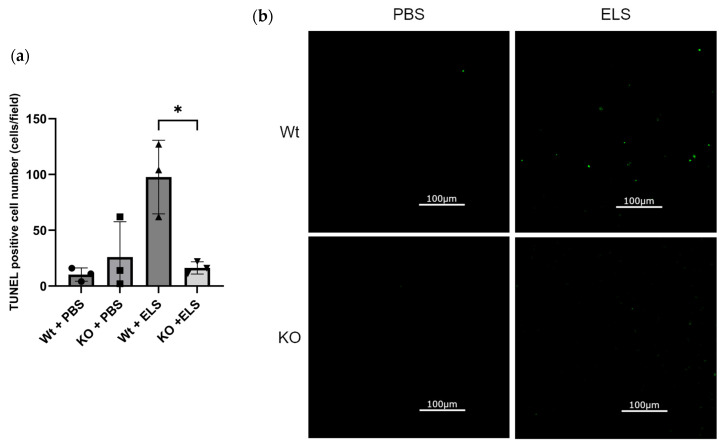

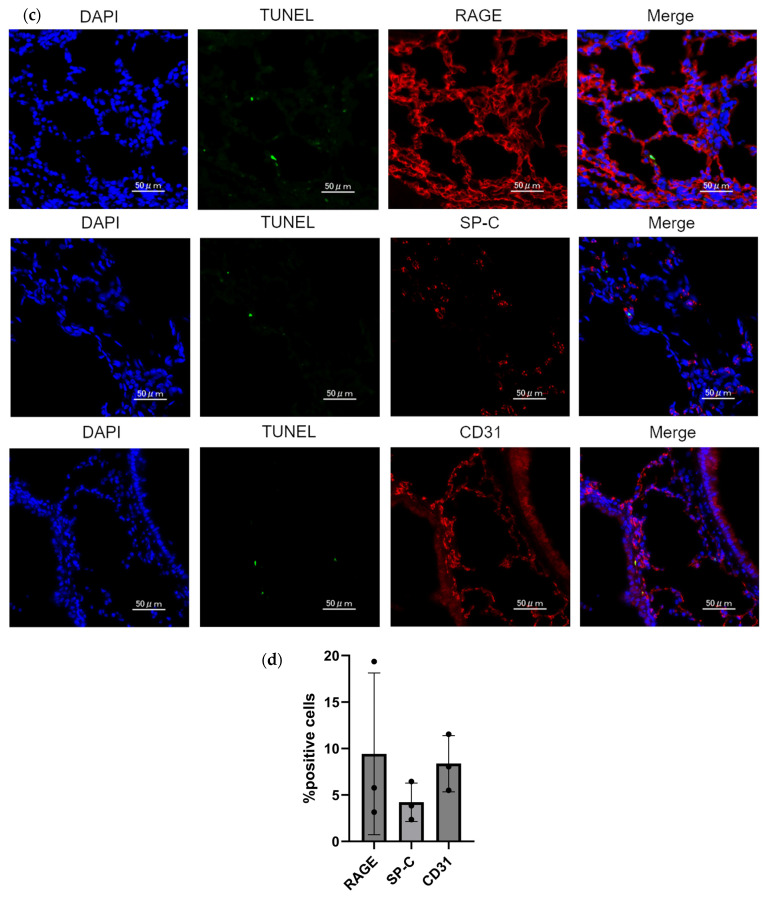
The extent and cell location of apoptosis in the lung on day 14. (**a**) TUNEL-positive cell count in lung tissue on day 14 (*n* = 3). (**b**) Representative TUNEL assay images showing lung tissues in each group. Scale bar, 100 µm. (**c**) Representative immunofluorescent staining of lungs for DAPI (blue), TUNEL (green), and RAGE (upper), SP-C (middle), or CD31 (lower) (red) in the Wt + ELS group is shown. Scale bar, 50 µm. (**d**) The percentage of RAGE-, SP-C-, and CD31-positive cells among TUNEL-positive cells in the Wt + ELS group is shown. * *p* < 0.05.

We evaluated apoptosis in the lung, considering that apoptosis is involved in the pathogenesis of emphysema and NETs are known to induce apoptosis in the lung. The number of TUNEL-positive cells was significantly increased in the Wt + ELS group compared to the KO + ELS group (Wt + ELS vs. KO + ELS; 97.7 ± 33.0 vs. 16.3 ± 5.5; *p* = 0.0135) (Figure 4a,b), suggesting that PAD4-KO deteriorates ELS-induced cell apoptosis in the lung. The TUNEL-positive alveolar epithelial type 1 (AT1) cells, alveolar epithelial type 2 (AT2) cells, and endothelial cells were identified in the Wt + ELS group using RAGE, SP-C, and CD31 immunostaining, suggesting that apoptosis was induced in AT1, AT2, and endothelial cells (Figure 4c).

Furthermore, to investigate whether PAD4 deficiency selectively protects specific lung cell populations, we performed quantitative immunofluorescence analysis of alveolar epithelial cells (type 1 and type 2) and endothelial cells in the Wt + ELS group. The proportions of RAGE, SP-C, and CD31 cells did not significantly vary among TUNEL-positive cells (Figure 4d). Due to the markedly reduced number of TUNEL-positive cells in PAD4-deficient mice, direct cell-type identification in this group was not performed. However, the significant decrease in overall TUNEL-positive cells in PAD4-deficient mice suggests that PAD4 deficiency broadly suppresses cell death rather than selectively protecting specific cellular populations.

### 2.5. RNA Sequencing and Real-Time Quantitative PCR Analysis of the Whole Lung

#### 2.5.1. RNA Sequencing on Day 1

**Figure 5:**
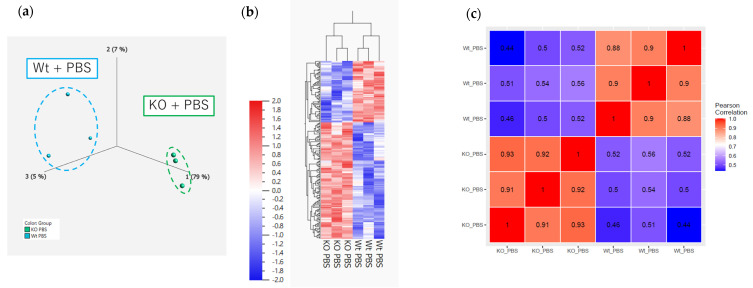

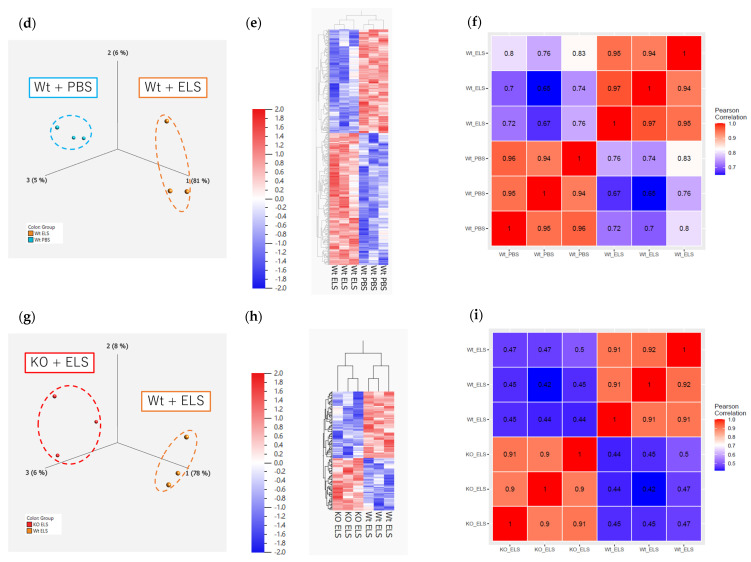
Principal component analysis, hierarchical clustering heatmap, and correlation heatmap of day 1. (**a**–**c**) The Wt + PBS group vs. the KO + PBS group (*n* = 3); (**d**–**f**) the Wt + PBS group vs. the Wt + ELS group (*n* = 3); (**g**–**i**) the Wt + ELS group vs. the KO + ELS group (*n* = 3).

To elucidate the role of PAD4 in the comprehensive gene expression signatures of ELS-induced lung inflammation, whole-lung RNA sequencing on day 1 and enrichment analysis (gene ontology (GO) and Wiki pathways) were performed. Principal component analysis and hierarchical clustering heatmap revealed that the gene expression patterns differed between the Wt + phosphate-buffered saline (PBS) and KO + PBS groups, the Wt + PBS and Wt + ELS groups, and the Wt + ELS and KO + ELS groups (Figure 5a,b,d,e,g,h), with 168, 595, and 116 identified DEGs, respectively (*p* < 0.05, fold change > 2). The correlation heatmap showed strong intra-group correlations, indicating high reproducibility and consistency among biological replicates (Figure 5c,f,i). These findings validate the reliability of the RNA sequencing data.

GO and pathway terms for the upregulated and downregulated genes in the KO + ELS and the Wt + ELS groups are listed in Table 1 and Table 2. The GO terms with elevated gene expression reflected “Negative Regulation Of ERK1 And ERK2 Cascade”, “Negative Regulation Of MAP Kinase Activity”, “Release Of Sequestered Calcium Ion Into Cytosol By Sarcoplasmic Reticulum”, and “Base-Excision Repair, AP Site Formation”; no Wikipathway terms with upregulated gene expression were identified (Table 1 and Table 2). The GO terms with downregulated gene expression reflected “Positive Regulation Of Lymphocyte Apoptotic Process”, “Proteolysis”, and “Regulation Of Platelet-Derived Growth Factor Receptor Signalling Pathway”; no Wikipathway terms with downregulated gene expression were identified.

Furthermore, we identified 12 genes that were upregulated in the Wt + ELS group compared to the Wt + PBS counterparts and downregulated in the KO + ELS group compared to the Wt + ELS counterparts; the top five of these genes included *ANGPTL7*, *PDCD1*, and *CNTN1* (Table 3).

#### 2.5.2. RNA Sequencing on Day 14

**Figure 6:**
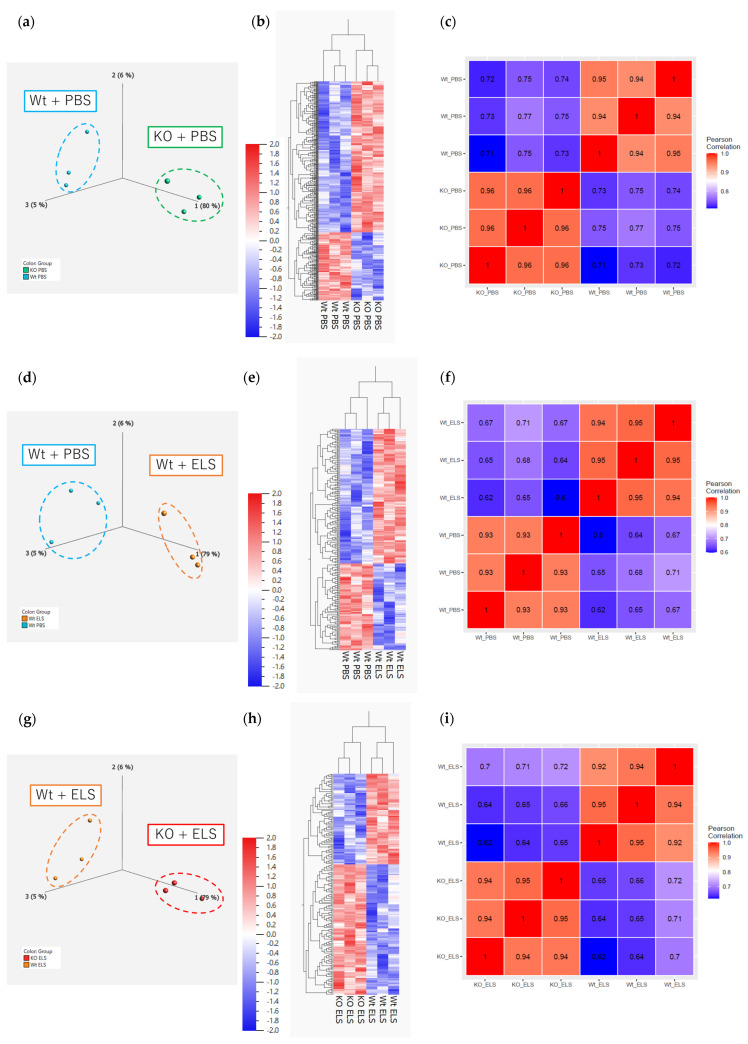
Principal component analysis, hierarchical clustering heatmap, and correlation heatmap of day 14. (**a**–**c**) The Wt + PBS group vs. the KO + PBS group (*n* = 3); (**d**–**f**) the Wt + PBS group vs. the Wt + ELS group (*n* = 3); (**g**–**i**) the Wt + ELS group vs. the KO + ELS group (*n* = 3).

Next, to elucidate the role of PAD4 in the comprehensive gene expression signatures of ELS-induced emphysema, RNA sequencing of the whole lung on day 14 and enrichment analysis were performed. Principal component analysis and a hierarchical clustering heatmap revealed that the gene expression patterns differed between the Wt + PBS and KO + PBS groups, the Wt + PBS and Wt + ELS groups, and the Wt + ELS and KO + ELS groups (Figure 6a,b,d,e,g,h), with 408, 219, and 183 identified DEGs, respectively (*p* < 0.05, fold change > 2). The correlation heatmap revealed strong intra-group correlations, indicating high reproducibility and consistency among biological replicates (Figure 6c,f,i). These findings validate the reliability of the RNA sequencing data.

GO and pathway terms for upregulated and downregulated genes in the KO + ELS compared to the Wt + ELS groups are listed in Table 4 and Table 5. The GO terms with elevated gene expression included “Regulation Of Cellular Extravasation”, “Entrainment Of Circadian Clock By Photoperiod”, and “Negative Regulation Of Phosphatidylinositol 3-Kinase Signalling”; moreover, Wikipathway terms with elevated gene expression included “Microglia Pathogen Phagocytosis Pathway”. The GO terms with downregulated gene expression included “Acute Inflammatory Response”, “Extracellular Matrix Organization”, and “Positive Regulation Of Cysteine-Type Endopeptidase Activity Involved In Apoptotic Signalling Pathway”; Wikipathway terms with downregulated gene expression included “Osteoblast Signalling”.

Furthermore, we identified 14 genes that were upregulated in the Wt + ELS group compared to the Wt + PBS group and downregulated in the KO + ELS group compared to the Wt + PBS counterparts; the top five of these genes included *HPGDS* (Table 6).

#### 2.5.3. Real-Time Quantitative PCR of the DEGs

**Figure 7:**
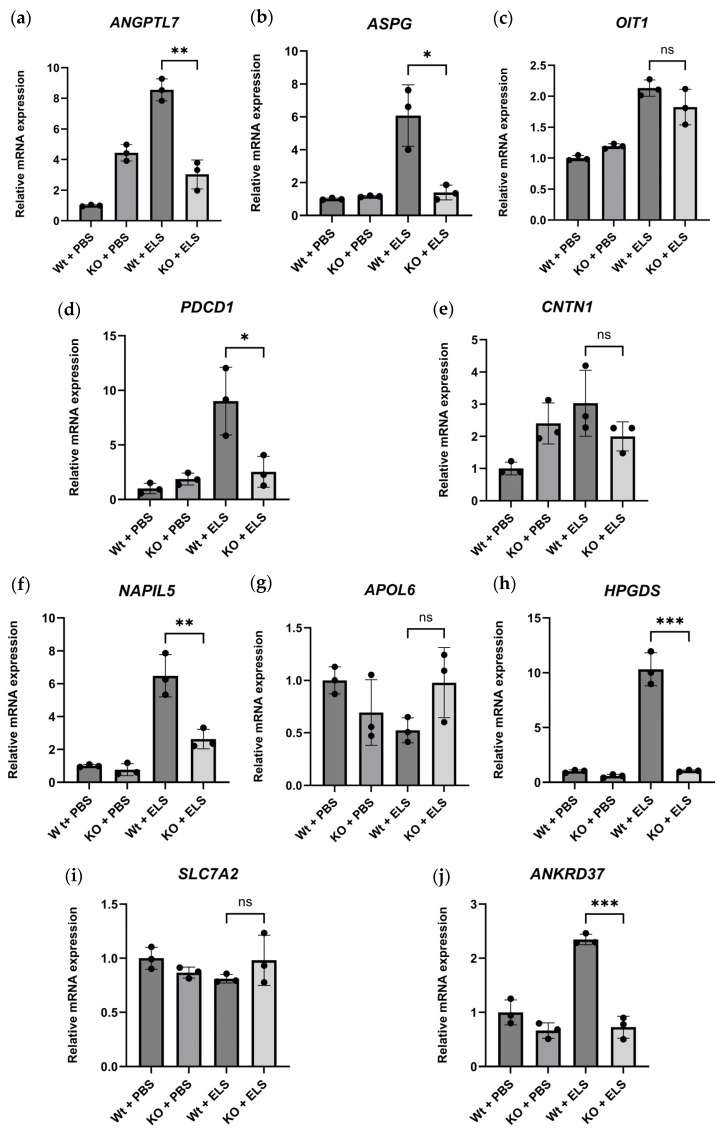
mRNA expression levels of the top five DEGs downregulated in the KO + ELS group compared to the Wt + ELS on day 1 (**a**–**e**), and day 14 (**f**–**j**). (*n* = 3). ns; not significant, * *p* < 0.05, ** *p* < 0.01. *** *p* < 0.001.

To validate the transcriptomic findings, we performed real-time quantitative PCR (qPCR) analysis of selected DEGs (Table 3 and Table 6). The qPCR results revealed gene expression profiles consistent with data acquired from the transcriptome analysis (Figure 7). Specifically, *ANGPTL7*, *ASPG*, *PDCD1*, *NAPIL5*, *HPGDS*, and *ANKRD37* were significantly downregulated by PAD4 deletion, presenting a similar trend to that detected in the RNA sequencing results. These findings support the robustness of our transcriptomic dataset and reinforce the biological relevance of the differential gene expression profiles.

## 3. Discussion

In the present study, we identified NETs in the lungs of mice with ELS-induced emphysema. After ELS treatment, NETs were observed in the interstitial space of the lungs, and PAD4 deficiency reduced the NET formation and tended to ameliorate tissue injury in the lung. PAD4 deficiency did not alter the inflammatory cellular influx to the alveolar region. Moreover, PAD4 deficiency ameliorated pulmonary emphysema and inhibited the apoptosis in lung cells. Finally, we revealed the effects of PAD4 on comprehensive gene expression signatures in ELS-induced lung inflammation and emphysema. Collectively, the results revealed an important role of PAD4 in ELS-induced NET formation and emphysema development.

Excessive NET release is associated with several inflammatory diseases [15,16,17], including lung fibrosis or COPD [18,20]. Previously, we reported that the deficiency of PAD4 ameliorates bleomycin (BLM)-induced formation of NETs and lung fibrosis in mice [18]. However, the role of PAD4 in ELS-induced emphysema remained unclear.

To the best of our knowledge, this is the first report that demonstrates the NET expression in the lung tissue of an ELS-induced emphysema mouse model. Previously, intratracheal ELS instillation was reported to cause neutrophil activation that induces excess ROS production in mice [26]. Moreover, ROS production is essential for NET formation [27,28]. Therefore, ROS production after ELS injection potentially stimulates NET formation. Additionally, histopathological analysis revealed that the damage to the lung tissue and NET formation was ameliorated by PAD4 depletion. These results suggest that PAD4 may play an important role in ELS-induced NET formation and tissue injury in the lung. Interestingly, the NET formation was partially blocked by PAD4 depletion. Although PAD4 plays a central role in NET induction among the five enzymes in the PAD family (PAD1, 2, 3, 4, and 6) [29], recent reports indicate that other factors, including PAD2, are involved in NET formation [30,31]. Thus, non-PAD4-mediated NET formation may have been induced in KO mice.

In the current study, the number and differentiation of BALF cells on days 1 and 14 did not significantly differ between the Wt and PAD4-KO groups, which suggests that PAD4 depletion does not affect the influx of inflammatory cells in the alveolar space during the acute and post-inflammatory (emphysema) phases of ELS-induced lung injury. Previous reports could not fully clarify the role of PAD4 in inflammatory cell influx into the lung. Previously, we demonstrated that the cell number and BLM-induced differentiation of BALF were not affected by PAD4 depletion [18]. Another group demonstrated that PAD4 deficiency did not affect the migration of white blood cells to the lung tissue in a mouse model of influenza [32]. Additionally, PAD4 deficiency does not affect the neutrophil influx in BALF; however, it inhibits NETosis, resulting in attenuated lung injury in a mouse model of phenylarsine oxide-induced ALI [33] or invasive pulmonary aspergillosis [34]. A lack of NETs may not be associated with a comprehensive reduction in neutrophil functions [35]. Hence, PAD4 may not play a critical role in the influx of inflammatory cells in the alveolar space in ELS-induced lung injury. However, further investigations can validate this possibility.

Here, we demonstrate that PAD4 deficiency ameliorates pulmonary emphysema. Several mechanisms, including abnormal inflammation [5], imbalance between proteolytic and anti-proteolytic activity [4], and oxidative stress [1,2,3,36], are involved in the development of COPD. Components of NETs, which include DNA, histones, and granule proteins, are known to induce cytotoxic and proinflammatory effects, leading to tissue injury [37]. In this study, PAD4 depletion may have contributed to the alleviation of tissue injury via inhibited NET formation, thereby preventing emphysema.

As mentioned above, we previously demonstrated that PAD4 deficiency alleviates BLM-induced NET formation in the lungs and significantly suppresses the degree of BLM-induced pulmonary fibrosis compared to wild-type mice. PAD4 deficiency prevents the decrease in alveolar epithelial and pulmonary vascular endothelial cell numbers and the increase in ACTA2-positive mesenchymal cells and S100A4-positive fibroblasts observed in the lung during fibrosis induced by BLM. The loss of NETs due to PAD4 deficiency may have a protective effect against these alterations in tissue cell components. PAD4 deficiency also suppressed the increased gene expression of inflammatory mediators induced by BLM during the acute phase of lung injury. Furthermore, by using bone marrow chimeric mice, we demonstrated that PAD4 expression in hematopoietic cells plays an important role in the development of BLM-induced pulmonary fibrosis. Collectively, these data suggest that PAD4-dependent NET formation is a significant contributor to the pathogenesis of lung fibrosis [18]. In a mouse model of COPD induced by chronic cigarette smoke exposure, administering the NE inhibitor, GW311616A, significantly impacted the disease pathology, suggesting that NET formation contributes to the cigarette smoke-induced lung injury. The study demonstrated that GW311616A administration substantially reduced pulmonary generation of NETs in the lungs of mice following chronic exposure to cigarette smoke. In addition, GW311616A showed a significant attenuation of key pathological changes associated with COPD induced by cigarette smoke exposure, including airway leukocyte infiltration, mucus-secreting goblet cell hyperplasia, and emphysema-like alveolar destruction. GW311616A treatment also attenuated the increased levels of neutrophil chemotactic factors (LTB4, KC, and CXCL5) and pro-inflammatory cytokines (IL-1β and TNF-α) in BALF and serum induced by cigarette smoke exposure. GW311616A treatment significantly improved indices of airflow limitation, such as FEV_1_/FVC, and inhibited the increase in indicators of emphysema, such as FRC, TLC, and FRC/TLC, in the COPD mouse model [38]. Collectively, PAD4-dependent NET formation might be a significant contributor to the pathogenesis of lung injury. NETs cause damage through direct cytotoxicity to lung cells, promoting inflammation, and contributing to detrimental tissue remodelling, like alveolar destruction and mucus hyperplasia. Therefore, reduction of NETs via inhibition of PAD4 or NE may serve as a promising strategy for the treatment of pulmonary emphysema and other NET-mediated lung injuries.

However, NE inhibitors do not specifically inhibit NETs and may suppress physiological NE functions. Furthermore, specifically targeting the activity of elastase is difficult owing to its functions overlapping with those of other serine proteases [39]. The results of the current study suggest that PAD4 inhibition may lead to the development of selective NET-targeting inhibitors for the treatment of COPD.

Additionally, we demonstrated that PAD4 contributes to a reduction in ELS-induced apoptosis in emphysematous lungs. Apoptosis in structural cells in the lungs is considered an important upstream event in the pathogenesis of COPD. Apoptosis in several types of cells, such as alveolar epithelial, endothelial, interstitial, and inflammatory cells, has been observed in the lungs of patients with COPD [40]. In recent years, components of NETs, MPO, and histones have been reported to potentially damage lung cells and cause cell death in lung endothelia and epithelia [21,41]. In the present study, ELS instillation induced apoptosis, which was suppressed by PAD4 depletion. In addition, we identified specific cell types in the Wt + ELS group because TUNEL-positive cells were significantly reduced in the KO + ELS group, making the direct comparison challenging. In the Wt mice, the proportions of alveolar epithelial cells and vascular endothelial cells did not significantly differ among TUNEL-positive cells. Previous studies report that increased cell death in both alveolar epithelial and endothelial cells is associated with the pathogenesis of emphysema [42,43]. Although we did not identify specific cell types in PAD4-deficient mice, the marked reduction in overall TUNEL-positive cells suggests that PAD4 deficiency may broadly suppress cell death rather than preferentially protecting a cell population. Further investigations incorporating quantitative immunofluorescence analysis of specific lung cell types can potentially refine our understanding of PAD4-related protective mechanisms.

Finally, we revealed the effect of PAD4 on comprehensive gene expression signatures on days 1 and 14.

On day 1, the expression of more than 200 genes varied after ELS instillation compared to that recorded in the PBS-treated group; furthermore, PAD4 depletion altered the expression profile of several hundred genes compared to Wt counterparts. Several GO terms and pathways associated with the pathogenesis of COPD were identified. PAD4 is associated with the downregulation of ‘negative regulation of mitogen-activated protein kinase (MAPK) activity’ related genes on day 1. The MAPK-ERK1/2 pathway was reported to be more activated in COPD lungs compared to healthy lungs [44]. Aberrant activation of MAP kinases contributes to several COPD-associated phenotypes, including mucus overproduction and secretion, inflammation, cytokine expression, apoptosis, T-cell activation, and matrix metalloproteinase production [45]. Dual-specificity phosphatases (DUSPs) are a family of proteins that can directly dephosphorylate MAPK proteins. DUSP10 was reported to negatively regulate inflammatory cytokines and promote the production of NO and ROS [46]. Moreover, a previous study revealed that tobacco smoke-induced NETs activate ERK signalling by releasing NE and MMP9 [47].

In a cigarette smoke-induced mouse model of COPD, impaired Ca^2+^ signals in intercostal and flexor digitorum brevis (FDB) muscle fibres were observed. Ryanodine receptor type-1 (RYR1) is crucial for Ca^2+^ release from the sarcoplasmic reticulum (SR), and RYR1 dysfunction may contribute to impaired Ca^2+^ signalling; the result of the study suggests that Ca^2+^ signalling dysregulation, potentially involving RYR1, may contribute to respiratory and locomotor muscle dysfunction in COPD [48]. However, the correlations between PAD4 and RYR1 remain unclear.

In addition, PAD4 is suggested to suppress the DNA repair process. Oxidative stress is evident in emphysema; moreover, free radical production and DNA damage are induced in pulmonary and adjacent cells [49]. As DNA repair and altered genomic stability are potentially involved in the cellular response to the elastase-induced acute lung injury [50] and excessive ROS generated during neutrophil activation causes widespread DNA damage and induces NET formation [27,51], PAD4 depletion may promote the DNA repair process by inhibiting NET formation.

The downregulated gene signatures on day 1 suggest a potential association of PAD4 depletion with “Positive Regulation Of Lymphocyte Apoptotic Process”. Programmed cell death protein 1 (*PDCD1*) was identified as a downregulated DEG. Treatment with anti-PD-1 reduced lung injury and neutrophilic inflammation in mice exposed to chronic cigarette smoke (CS) [52]. To our knowledge, the association of PAD4 with PD-1 remains unknown. “Proteolysis” suggests that the destruction of the extracellular matrix and other components of the lung, which is caused by inflammation and emphysema, is suppressed compared to the Wt + ELS group than the KO + ELS group. In particular, a disintegrin and metalloprotease with thrombospondin motifs (ADAMTS) family, including *ADAMTS12*, acts as a protease that regulates the composition of the extracellular matrix. Platelet-derived growth factor (PDGF) signalling is crucial for lung development and associated diseases, including emphysema [53]. The deficiency of PDGF-A in mice causes pulmonary emphysema secondary to defective formation of alveolar septa induced by defective differentiation of alveolar myofibroblasts [53,54].

Moreover, we detected several common genes in the Wt + ELS group compared to the Wt + PBS group that were downregulated in the Wt + ELS group compared to the KO + ELS group; these include *ANGPTL7*, *PDCD1*, and *CNTN1*. *ANGPTL7* codes a component of the extracellular matrix. In this study, the ELS instillation caused the formation of emphysema, leading to the destruction of the extracellular matrix. Therefore, it is thought that the genes involved in extracellular matrix formation may increase in response to the ELS-induced lung injury, and this reaction may promote tissue repair.

ELS instillation altered the expression of more than 400 genes on day 14 compared to that in the PBS treatment group; moreover, the PAD4 depletion affected the expression of more than 180 genes compared to their Wt counterparts. In particular, GO terms with elevated gene expression included “Regulation Of Cellular Extravasation”, “Entrainment Of Circadian Clock By Photoperiod”, and “Negative Regulation Of Phosphatidylinositol 3-Kinase Signalling”. Furthermore, Wikipathway terms with elevated gene expression included “Microglia Pathogen Phagocytosis Pathway”.

Among “Regulation Of Cellular Extravasation”, *PLVAP* and *LYVE1* were upregulated DEGs. Plasmalemmal vesicle-associated protein-1 (PLVAP) is essential for generating spoke-like diaphragmatic structures that span the neck region of endothelial caveolae. PLVAP in caveolae controls lung endothelial permeability and is required for the maintenance of lung vascular-barrier integrity [55].

Lymphatic vessel endothelial hyaluronan receptor 1 (LYVE1), mainly expressed in the lymphatic endothelium, is associated with pulmonary lymphangiogenesis. LYVE1 expression is altered in COPD and it regulates an adaptive immune response and airway remodelling [56,57]. Additionally, NETs are expressed in mouse models of foreign body reactions to silicon; LYVE1-positive macrophages and mast cells potentially contribute to the regulation of inflammation [49]. Hence, PAD4 deletion possibly improves lymphatic and vascular maintenance in ELS-induced emphysema in mice.

Disrupted daily or circadian rhythms of lung function and inflammatory responses are common in chronic airway diseases, including COPD [58,59,60]. *CRY1* encodes a flavin adenine dinucleotide-binding protein cryptochrome 1 (CRY1), a major component of the circadian core oscillator complex that controls the circadian clock; the expression of this gene was reported to be suppressed in smokers [61]. Environmental agents, including cigarette smoke and redox modulation, may alter the levels of clock gene expressions in the lung along with a heightened DNA damage response, cellular senescence, and inflammation [60]. The role of CRY1 and its relationship with PAD4 remain unclear; however, its dysregulation potentially contributed to the enhanced inflammation and altered lung injury observed in the present study.

In addition, the NOD-like receptor family CARD domain containing 3 (NLRC3) is considered a negative regulator of inflammation; it inhibits the formation of the NOD-like receptor family pyrin domain containing 3 (NLRP3) inflammasome. The NLRP3 inflammasome, a multi-protein complex, is associated with COPD [62]. The activated NLRP3 inflammasome regulates the release of inflammatory factors, leading to chronic inflammation and tissue damage. Recent studies revealed that NETs can activate the NLRP3 inflammasome, leading to inflammation and thrombosis [63]. Interestingly, the NLRP3 inflammasome assembly in neutrophils is supported by PAD4 and promotes NETs [64]. The results of this study suggested that the expression of NLRC3 was upregulated and the activity of NLRP3 was suppressed by the inhibition of ELS-induced NETs by PAD4-KO.

We identified upregulation of “Microglia Pathogen Phagocytosis Pathway”, and *LAT* and *SIGLECE* were identified as DEGs. Sialic acid-binding immunoglobulin-like lectin (SIGLEC) 9 is a member of the SIGLEC cell surface immunoglobulin family, and SIGLECE is a functional homologous molecule in mice. Previous reports demonstrate that anti-SIGLECE antibodies suppress neutrophil recruitment and activation in a mouse model of airway inflammation [65]. Furthermore, soluble SIGLEC9 (sSIGLEC9), the extracellular domain of SIGLEC9, may partially fulfil its function by competitively inhibiting the binding of SIGLEC9 to its ligands. SIGLEC9 was reported to possibly induce negative feedback by increasing complementarily, which limits neutrophil activation in COPD; moreover, it competitively inhibits the ligands that bind to SIGLEC9 [66]. These mechanisms may underlie the outcomes of the current study; however, the precise mechanisms need further investigation.

The downregulated gene signatures on day 14 suggest a potential association of PAD4 depletion with “Acute Inflammatory Response”. Previously, in patients with COPD, exposure to particulate matter ≤ 10 µm (PM10) was reported to increase inter-alpha-trypsin inhibitor heavy chain 4 (ITIH4) levels in correlation with CRP; moreover, ITIH4 may be involved in inflammatory mechanisms [67]. In the current study, PAD4-KO suppressed ITIH4, suggesting its involvement in ELS-induced inflammation. Additionally, “Extracellular Matrix Organization” was identified as a GO term. *LOX* encodes lysyl oxidase. Previous reports reflect the correlation of LOX with smoking and its contribution to airway dysfunction [68]. *ELN* encodes elastin, one of the two components of elastic fibres, and an extracellular matrix protein (ECM) that supports lung elasticity. Structural changes in elastin reduce lung functions in COPD patients, which is considered to be related to an uncontrolled activation of proteases, including NE, in the airways. These proteases specifically target elastin, and the loss of elastin leads to the collapse of small airways, ultimately causing emphysema [69]. NETs contain several granular enzymes, including NE, which promote extracellular matrix degradation. Therefore, increased extracellular matrix organization in Wt mice may be a part of the repair response to emphysema.

In “Positive Regulation Of Cysteine-Type Endopeptidase Activity Involved In Apoptotic Signalling Pathway”, *ST18* was identified as a DEG. Tumorigenicity 18 (ST18) was reported to mediate the mRNA levels of genes that promote apoptosis and inflammation induced by tumour necrosis factor (TNF)-α in fibroblasts [70]. These findings appear consistent with those of our TUNEL assay.

Furthermore, we detected common genes that were upregulated in the Wt + ELS group compared with the Wt + PBS counterparts and downregulated in the KO + ELS group compared with the Wt + ELS counterparts, which included *HPGDS*. *HPGDS* encodes hematopoietic prostaglandin D synthase (HPGDS), mainly found in mast cells; moreover, PGD2 acts as a mediator in allergic asthma [71]. In COPD subjects, PGD2 was negatively correlated with airway obstruction. COPD subjects with disease exacerbations had significantly higher concentrations of PGD2 [72]. Pharmacological blockade of the DP2 receptor inhibits cigarette smoke-induced inflammation, mucus cell metaplasia, and epithelial hyperplasia in the mouse lung [73]. Therefore, the reduction of HPGDS may be associated with the alleviated ELS-induced lung inflammation and injury.

To strengthen the reliability of our transcriptomic data, we conducted qPCR analysis on selected differentially expressed genes. The qPCR results confirmed the trends observed in our transcriptome analysis, reinforcing the robustness of the gene expression findings. Specifically, the expression patterns of *ANGPTL7*, *ASPG*, *PDCD1*, *NAPIL5*, *HPGDS*, and *ANKRD37* exhibited consistency across both approaches, demonstrating the validity of our dataset. These findings support the notion that our transcriptomic analysis accurately reflects gene regulation under the experimental conditions. While our validation confirms the overall reliability of our transcriptomic dataset, further studies are warranted to elucidate the functional implications of differentially expressed genes. Investigating their downstream pathways and regulatory mechanisms could provide additional insights into the molecular processes influenced by PAD4 deficiency.

The clinical feasibility of PAD4 inhibitors as therapeutic agents for COPD remains in the early stages of investigation, driven by a strong scientific rationale rather than established clinical efficacy in COPD specifically. PAD4-mediated protein citrullination and NET formation are implicated in various diseases [74]. PAD4 inhibitors suppress citrullination and NET formation [74], making them a potential strategy for mitigating NET-associated pathology in several conditions, including COPD. However, most research is still at the experimental or preclinical stage, and no PAD4 inhibitor currently meets the necessary criteria for clinical use, such as high potency and selectivity [74].

One major challenge is the potential off-target effects due to the high structural conservation among PAD isozymes, which complicates selective inhibition [75]. Early PAD inhibitors were non-selective pan-PAD inhibitors [76]. Although newer compounds exhibit improved selectivity for PAD4 over other isoforms (PAD1, PAD2, PAD3, PAD6) [74], off-target inhibition of these isozymes—each with distinct tissue distributions and physiological roles—is generally undesirable [75].

Moreover, PAD4 inhibition may exhibit synergistic effects when combined with existing treatments, including neutrophil elastase inhibitors, DNase therapy, and broad-spectrum anti-inflammatory agents. A combinatorial approach incorporating PAD4 inhibitors could enhance therapeutic outcomes by concurrently suppressing NET formation, excessive neutrophilic activity, and chronic inflammatory burden.

To improve the translational relevance and clinical impact of PAD4 inhibition in COPD, further efforts are needed to develop highly potent and selective PAD4 inhibitors while conducting rigorous preclinical studies in appropriate models. Investigating potential synergies with existing therapies may reveal beneficial combination strategies; however, COPD-specific studies assessing these interactions remain necessary.

This study has several limitations. First, the ELS-induced emphysema model cannot reproduce smoking-induced emphysema formation. Creating a cigarette-smoking-induced emphysema mouse model involves time-consuming processes, and hence, we decided to use the ELS-induced emphysema mouse model in this study. Although animal models provide key insights into NET-mediated lung injury, the direct applicability of our findings to human COPD requires further validation. To address this limitation, future studies should incorporate clinical investigations and patient-derived samples to confirm the translational relevance of our results. Previous reports have demonstrated similarities between murine and human COPD pathophysiology [77], supporting the biological significance of our findings; however, additional work is needed to assess whether PAD4 inhibition and NET-targeting strategies yield comparable effects in human disease contexts. Despite these limitations, this study provides critical mechanistic insights into NET-driven lung injury and serves as a foundation for subsequent translational research. The findings contribute to the ongoing effort to understand NET pathology in COPD and inform the development of potential therapeutic approaches tailored for human patients.

Second, in this study, the small sample size (*n* = 3–6 for BALF and lung tissue pathology, and *n* = 3 for RNA sequencing) limits the statistical robustness of data and their suitability to be generalised; moreover, genes with small differences in expression levels may not be identified. However, the gene expression profiles were highly consistent within individual groups and significantly distinguishable between the Wt and KO groups. Principal component analysis revealed that the first principal component accounted for over 70% of the total variance and effectively separated the two groups. Furthermore, principal component analysis and hierarchical clustering heatmap revealed distinct expression patterns between the two groups. The correlation heatmap analysis also demonstrated a high degree of transcriptomic similarity within each experimental group. Additionally, qPCR analysis of selected differentially expressed genes further validated the transcriptomic data. qPCR results were consistent with the expression patterns observed in the high-throughput analysis, further supporting the reliability and biological relevance of the findings. These findings suggest strong consistency among biological replicates and support the reliability of the transcriptomic findings. The transcriptomic analysis revealed significant dysregulation in pathways implicated in emphysema pathogenesis, including MAPK signalling, DNA damage response, neutrophil activation, inflammation, protease activity, extracellular matrix remodelling, NLRP3 inflammasome activation, and TNF-α signalling [45,49,62]. Previously, these pathways have been extensively documented in emphysema and chronic lung disease studies, reinforcing the relevance of our results. Furthermore, elastase-induced lung inflammation and emphysema-related pathological features were consistent with previous reports [49,78,79]. Notably, KO mice exhibited a marked reduction in alveolar destruction, which corresponded with the suppression of emphysema-associated gene expression identified in RNA sequencing. The concordance between transcriptomic and histological data strengthens the robustness of our conclusions. Nevertheless, further validation of these findings using larger cohorts can strengthen the generalizability of the conclusions.

Thirdly, the results of this study are potentially associated with not only NETs but also some unexplored functions of PAD4. For example, elastin is a target of citrullination by PAD enzymes, and it has been reported that citrullinated elastin levels in the peripheral airways are significantly higher in patients with COPD than in healthy individuals. Additionally, when PAD inhibitors are administered to mice, PAD-induced citrullination of elastin is inhibited, and the formation of emphysema is suppressed [80]. Finally, although our results provide an aspect of the pathophysiological concept of NET expression and ELS-induced pulmonary emphysema, the DEGs identified in this study require further independent validation.

## 4. Materials and Methods

### 4.1. Mice and ELS-Induced Emphysema Model

Male C57BL/6 mice were purchased from Japan SLC (Shizuoka, Japan) and CLEA Japan (Tokyo, Japan). PAD4—mice with a C57BL/6 background were purchased from Jackson Laboratory (Bar Harbor, ME, USA). All animal experiments were reviewed and approved by the Review Board for Animal Experiments of Chiba University. Pancreatic porcine ELS was purchased from Elastin Products Company (Owensville, MO, USA, cat. EC134). ELS was dissolved in glycerol and 50% 0.02 M NaOAc pH5 and stored at −20 °C. At the time of use, ELS was adjusted to 3 U/100 µL with PBS. The 812-week-old mice were used in the study. Intratracheal administration of 3 U/100 µL ELS (ELS group) and 100 µL PBS (the control group) was performed, and animals were categorised into two groups accordingly.

### 4.2. BALF Analysis

Following the anaesthetisation of the animals using medetomidine, midazolam, and butorphanol, BALF was collected by injecting 500 mL of PBS into the lungs through a tracheal cannula three times 1 and 14 days after the ELS administration. We collected cells from BALF via centrifugation (4 °C, 300× *g*, 5 min). The total cell counts and cell fractions were measured using an automatic cell counter (Countess II FL Automated Cell Counter; Thermo Fisher Scientific, Waltham, MA, USA) and the cytospin method, respectively.

### 4.3. Evaluating Emphysema Formation

The left lung was fixed using 4% paraformaldehyde for 20 min, washed with H_2_O, and paraffin-embedded. Subsequently, 5 μm slices of the lung were stained using Hematoxylin and Eosin. We calculated the mean linear intercept (Lm) and acute lung injury (ALI) scores using the haematoxylin and eosin-stained samples on day 14. Lm was calculated considering 20 zones per sample following a previously reported method [81] using a Nikon ECLIPSE 55i microscope (Nikon, Tokyo, Japan). The ALI score was calculated using a microscope Olympus CH30 (Olympus, Tokyo, Japan) as previously described [24].

### 4.4. Detection of NETs in the Lung

We evaluated the ELS-induced expression of NETs using fluorescence immunostaining following previously reported protocols [25]. For this analysis, 5 μm paraffin sections were prepared and deparaffinised; next, slides were hydrated by separately immersing them in xylene and 100% ethanol for 15 min each. To activate the antigen, citrate buffer (pH 6.0) was added and incubated at 100 °C for 25 min. After blocking the samples by treating them with 1% bovine serum albumin (BSA)/PBS for 25 min at room temperature, the slides were incubated overnight with the following antibodies at 4 °C: goat polyclonal anti-myeloperoxidase (anti-MPO) (1:200; R&D Systems, Minneapolis, MN, USA, cat. AF3667) and rabbit polyclonal anti-histone H3 (1:100; Abcam, Cambridge, UK; cat. ab281584). The slides were washed with 0.5% Tween 20 and incubated with donkey anti-goat IgG Alexa Fluor 488 (1:500; Abcam, Cambridge, UK; cat. ab150129), used as the secondary antibody, and alpaca anti-rabbit IgG Alexa Fluor 594 (1:500; Jackson ImmunoResearch, West Grove, PA, USA, cat. 611-585-215). Next, the slides were mounted using DAPI 2HCl (Tokyo Chemical Industry, Tokyo, Japan, cat. A2412). For each sample, the number of MPO^+^ cells and frequency of CitH^3+^ cells among the MPO^+^ cells were calculated based on 10 fields of view at 200 high magnification, and the CitH3^+^ in MPO^+^ cells ratio was calculated considering the average values. Outliers were excluded considering the interquartile range. Images were acquired using a KEYENCE BZ-X710 (KEYENCE, Osaka, Japan) and analysed using Fiji with ImageJ version 1.54 f (National Institutes of Health, Bethesda, MD, USA).

### 4.5. SEM Analysis

SEM analysis was performed 14 days after ELS instillation. For this analysis, 100 μm paraffin sections were prepared. After deparaffinization, the cells were fixed using 2.5% glutaraldehyde and treated with a 2.5% phosphotungstic acid solution for conductive treatment. The samples were observed using a low-vacuum SEM TM3030 (Hitachi High-Tech Corporation, Tokyo, Japan).

### 4.6. TUNEL Assay

To evaluate apoptosis, we performed TUNEL staining on day 14 using the DeadEnd Fluorometric TUNEL System (Promega, Fitchburg, WI, USA, cat. G3250) following the protocol provided by the manufacturer. We used rabbit anti-Prosurfactant Protein C antibody (1:1170; Abcam, Cambridge, UK, cat. ab90716), anti-RAGE antibody (1:4000; Abcam, Cambridge, UK, cat. ab228861), anti-CD31 antibody (1:2000; Abcam, Cambridge, UK, cat. ab182981), and alpaca anti-rabbit IgG Alexa Fluor 594 (1:500; Jackson ImmunoReseach, West Grove, PA, USA, cat. 611-585-215) for co-staining. The antibody dilution solution was 1% BSA/PBS. For combined staining of SP-C and RAGE with TUNEL, antigen retrieval was performed using citrate buffer after proteinase K treatment. For combined staining of CD31 and TUNEL, retrieval was performed using a Tris-EDTA buffer. After immunofluorescence staining, TUNEL staining was performed following the manufacturer’s instructions. Nuclear staining was performed using DAPI 2HCl (Tokyo Chemical Industry, Tokyo, Japan, cat. A2412). The number of TUNEL-positive cells was calculated based on the average values derived from 10 fields analysed at 100 high magnification in each sample. Images were acquired using KEYENCE BZ-X710 (KEYENCE, Osaka, Japan).

### 4.7. RNA Sequencing

We used the right lung stored at −80 °C after resection with Allprotect Tissue Reagent (QIAGEN, Venlo, The Netherlands, cat. 76405) to purify RNA using the RNeasy Plus Mini Kit (QIAGEN, Venlo, The Netherlands, cat. 74134) according to the manufacturer’s protocol. The quality and concentration of the RNA were verified using a Qubit fluorometer (Life Technologies, Carlsbad, CA, USA) and an Agilent 2100 bioanalyzer (Agilent Technologies, Santa Clara, CA, USA). Purified total RNA (500 ng) was used to prepare RNA libraries using the Quant Seq 3′ mRNA-seq library preparation kit FWD of Illumina (LEXOGEN, Vienna, Austria) following the instructions provided by the manufacturer. RNA libraries were sequenced on an Illumina NextSeq 500 system with 75-nt-long reads.

The 3′ RNA-sequencing data were processed using Perseus software ver. 1.6.15.0 (Max Planck Institute of Biochemistry, Bayern, Germany). The RPM data were log2 transformed and filtered to ensure each gene contained at least 70% valid values in at least one group. The remaining missing values were supplemented with random numbers drawn from a normal distribution (width = 0.3, shift = 2.8). The unpaired Student’s *t*-test was used to compare the two groups. Statistical significance was defined as a two-tailed *p*-value < 0.05. We calculated the false discovery rate, treated it as a q-value, and considered it while interpreting the *p*-value. Moreover, 3′ RNA sequencing RPM data was analysed using Qlucore Omics Explorer software ver. 3.9.9 (Qlucore AB, Lund, Sweden); this software for data analysis and mining is based on mathematical and statistical methods (general linear statistical models based on R). The fold change between groups was greater than 2.0 (*p* < 0.05). Significantly upregulated or downregulated functional categories were identified using the Enrichr online tool “http://amp.pharm.mssm.edu/Enrichr/” (accessed on 13 February 2025). The gene set databases used in this study were “GO_Biological_Process_2023” (Terms: 5407; gene coverage: 14698) and “WikiPathways_2024_Mouse” (Terms: 188; gene coverage: 4494). GO terms and Wikipathways were also identified and considered significant at *p* < 0.05.

### 4.8. Real-Time Quantitative PCR Analysis

The total RNA was extracted from the whole mouse lung, as described above. The extracted RNA was reverse transcribed via PCR using the SuperScript IV VILO Master Mix (Thermo Fisher Scientific, Waltham, MA, USA) to synthesize single-stranded cDNA. cDNA samples were amplified through qPCR on the GeneAmp PCR System (Thermo Fisher Scientific, Waltham, MA, USA) using the Fast SYBR Green PCR Master Mix (Thermo Fisher Scientific, Waltham, MA, USA). The primer sequences utilized in this analysis were as follows: *ANGPTL7*, F: 5′-ACACCGTCTTCAGCACCAAG-3′, R: 5′-TACCAGTAGCCACCTTTTCGG-3′, *ASPG*, F: 5′-ATCAAGGGGAGACAGCGAAG-3′, R: 5′-CAGCTGCTTCAGCTACTTGC-3′, *OIT3*, F: 5′-ACTTTGGCATAGAGCCCCTG-3′, R: 5′-TCATCGGAAACACAGCCGTC-3′, *PDCD1*, F: 5′-CGGTTTCAAGGCATGGTCATTGG-3′, R: 5′-TCAGAGTGTCGTCCTTGCTTCC-3′, *CNTN1*, F: 5′-ACTCTGCACAAGATGCTCCC-3′, R: 5′-CCAGCCCAGTACCGAATCTG-3′, *NAP1L5*, F: 5′-TGCGTCACACGTTGAAATCC-3′, R: 5′-ATGACGTCGTTCCTGGGTTC-3′, *APOL6*, F: 5′-AGGACTCTTGGAGAGGGAGG-3′, R: 5′-TGTCTGGAAGGATTACCGTGC-3′, *HPGDS*, F: 5′-GAATAGAACAAGCTGACTGGC-3′, R: 5′-AGCCAAATCTGTGTTTTTGG-3′, *SLC7A2*, F: 5′-GTGTGCCTTGTATTACTCCTGGC-3′, R: 5′-CCACCATGACAAAGAGAAGGACC-3′, *ANKRD37*, F: 5′-AGCATCAGTGAACGCACCTCCG-3′, R: 5′-ACCTTTGCTGCCTTGTGGAGTG-3′, *HPRT1*, F: 5′-CCCAGCGTCGTGATTAGTGATG-3′, R: 5′-TTCAGTCCTGTCCATAATCAGT-3′.

The expression levels of target genes were normalized to threshold cycle (CT) values of hypoxanthine phosphoribosyl transferase 1 and were calculated using the 2^−∆∆Ct^ method (∆∆CT = [target gene CT of experimental group-reference gene CT of experimental group] − [target gene CT of control group-reference gene CT of control group]).

### 4.9. Statistical Analysis

Statistical analyses were performed using GraphPad Prism ver. 10.3.1 (GraphPad Software, Boston, MA, USA). Data is presented as the mean ± standard deviation, and unpaired two-tailed *t*-tests were used for comparisons of the two groups. Comparisons among the three groups were made using a repeated-measures one-way ANOVA with the Geisser–Greenhouse correction. Statistical significance was set at *p* < 0.05.

## 5. Conclusions

In conclusion, this study reveals that NETs are induced by ELS instillation and that PAD4 deficiency supresses ELS-induced neutrophil extracellular traps and emphysema in mouse lungs, which are associated with reduced apoptosis in the lung. Furthermore, we demonstrated the effect of PAD4 on comprehensive gene expression signatures associated with emphysema pathogenesis. Collectively, our results suggest that inhibition of PAD4 can be considered a potential therapeutic target for the treatment of COPD.

## Figures and Tables

**Table 1:** Upregulated genes in the KO + ELS groups compared to Wt + ELS counterparts, as identified through enrichment analysis of transcriptomic data on day 1.

GO: Relevant Terms Were Excerpted		
GO Terms	DEGs	*p*-Value
Negative Regulation Of ERK1 And ERK2 Cascade (GO:0070373)	*DUSP10 RGS14*	0.0067
Negative Regulation Of MAP Kinase Activity (GO:0043407)	*RGS14*	0.0076
Release Of Sequestered Calcium Ion Into Cytosol By Sarcoplasmic Reticulum (GO:0014808)	*RYR1*	0.015
Base-Excision Repair, AP Site Formation (GO:0006285)	*MUTYH*	0.028

**Table 2:** Downregulated genes in the KO + ELS group compared to Wt + ELS counterparts, as identified through enrichment analysis of transcriptomic data on day 1.

GO: Relevant Terms Were Excerpted		
GO Terms	DEGs	*p*-Value
Positive Regulation Of Lymphocyte Apoptotic Process (GO:0070230)	*PDCD1*	0.019
Proteolysis (GO:0006508)	*DDI2 HTRA4 CNTN1 ADAMTS12*	0.022
Regulation Of Platelet-Derived Growth Factor Receptor Signalling Pathway (GO:0010640)	*CNTN1*	0.038

**Table 3:** The top five DEGs upregulated in the Wt + ELS group compared to Wt + PBS counterparts and downregulated in the KO + ELS group compared to Wt + ELS counterparts on day 1.

DEGs	Difference (Wt + PBS vs. Wt + ELS) (Fold Change)	Difference (Wt + ELS vs. KO + ELS) (Fold Change)
1. *ANGPTL7*	4.06	−1.96
2. *ASPG*	2.93	−2.96
3. *OIT3*	2.39	−3.46
4. *PDCD1*	2.74	−2.56
5. *CNTN1*	3.02	−1.77

**Table 4:** Upregulated genes in the KO + ELS groups compared to Wt + ELS counterparts, as identified through enrichment analysis of transcriptomic data on day 14.

GO: Relevant Terms Were Excerpted		
GO Term	DEGs	*p*-Value
Regulation Of Cellular Extravasation (GO:0002691)	*PLVAP LYVE1*	0.0011
Entrainment Of Circadian Clock By Photoperiod (GO:0043153)	*USP2 CRY1*	0.0070
Negative Regulation Of Phosphatidylinositol 3-Kinase Signalling (GO:0014067)	*NLRC3*	0.048
**Wikipathways: Relevant Pathways Were Excerpted**		
WikiPathway	DEGs	*p*-value
Microglia Pathogen Phagocytosis Pathway (WP3626)	*LAT SIGLECE*	0.021

**Table 5:** Downregulated genes in the KO + ELS group compared to Wt + ELS counterparts, as identified through enrichment analysis of transcriptomic data on day 14.

GO: Relevant Terms Were Excerpted		
GO Term	DEGs	*p*-Value
Acute Inflammatory Response (GO:0002526)	*ITIH4 VNN1*	0.0043
Extracellular Matrix Organization (GO:0030198)	*LOX ELN ADAMTS6 COL19A1*	0.0095
Positive Regulation Of Cysteine-Type Endopeptidase Activity Involved In Apoptotic Signalling Pathway (GO:2001269)	*ST18*	0.022
**Wikipathways: Relevant Pathways Were Excerpted**		
WikiPathway	DEGs	*p*-value
Osteoblast Signalling (WP238)	*SLC17A2*	0.037

**Table 6:** The top five DEGs upregulated in the Wt + ELS group compared to Wt + PBS counterparts and downregulated in the KO + ELS group compared to Wt + ELS counterparts on day 14.

DEGs	Difference (Wt + PBS vs. Wt + ELS) (Fold Change)	Difference (Wt + ELS vs. KO + ELS) (Fold Change)
1. *NAP1L5*	3.18	−2.39
2. *APOL6*	3.19	−2.25
3. *HPGDS*	3.56	−1.87
4. *SLC7A2*	2.62	−2.19
5. *ANKRD37*	1.25	−3.12

## Data Availability

The datasets presented in this study can be found online in the NCBI database (accession number: GSE284684).

## Annotation

This article is a revised and expanded version of a presentation entitled “The role of neutrophil extracellular traps in the pathogenesis of elastase-induced emphysema in mice”, which was presented at the 64th Annual meeting of the Japanese Respiratory Society in Yokohama, Japan, 5 April 2024, and the European Respiratory Society Congress 2024 in Vienna, Austria, 6 September 2024.
